# Recurrence of a Cellular Blue Nevus with Satellitosis—A Diagnostic Pitfall with Clinical Consequences

**DOI:** 10.3390/dermatopathology9040042

**Published:** 2022-11-15

**Authors:** Hermann Kneitz, Christian Rose, Valerie Glutsch, Matthias Goebeler

**Affiliations:** 1 Department of Dermatology, Venereology and Allergology, University Hospital Würzburg, 97080 Würzburg, Germany; 2 Dermatopathology Laboratory, 23562 Lübeck, Germany

**Keywords:** common blue nevus, cell rich blue nevus, satellitosis, immunohistochemistry, skin

## Abstract

Blue nevus is a benign melanocytic lesion, typically asymptomatic and of unknown etiology. Several histologic and clinical variants have been distinguished, the most frequent being common blue nevus, cellular blue nevus, and combined blue nevus. Although melanocytic nevi with a satellite lesion are usually suggestive of locally advanced malignant melanoma, very few cases of blue nevi with satellite lesions have been reported. The diagnosis of common or cellular blue nevi is generally straightforward; however, the presence of structures such as irregular edges or satellitosis are highly suggestive for malignancy, and differential diagnoses such as locally advanced malignant melanoma and malignant blue nevus should be considered. Recurrent blue nevi can display atypical features not seen in the primary lesion, such as pleomorphism and mitotic activity. They usually tend to follow a benign course; however, in some cases, recurrence may indicate malignant transformation. We here report the unique case of a 64-year-old woman with a recurrent cellular blue nevus accompanied by satellite lesions. Such a biological behavior resulting in a clinical presentation as a melanoma-like lesion is a rarity in blue nevus and has not been described before.

## 1. Introduction

The blue nevus (BN) is a rather unusual, acquired benign melanocytic lesion of the skin. It is caused by the migration of melanocytes from the neural crest into the epidermis and deep subcutaneous tissue. Histopathological features show a dark pigmented tumor composed of dendritic and spindled melanocytes. Cellular blue nevus (CBN) is a pigmented biphasic tumor with a component of classic blue nevus and distinct cellular areas of spindled to oval melanocytes with clear or finely pigmented cytoplasm. The diagnosis of BN or CBN is usually not difficult. However, in a very few number of cases, including our case, a BN or CBN can accompanied by satellite lesions [1,2,3,4,5,6,7,8,9,10].

Recurrent melanocytic nevus is defined as the proliferation of remaining melanocytes arising from a partially removed melanocytic nevus, which leads to repigmentation at the site of a previous excision. Shave excisions are often related to recurring nevi. In the majority of the cases, recurrent melanocytic nevi display asymmetric borders and irregular pigmentation, which may lead to diagnostic confusion with melanoma. Recurrence of common melanocytic nevi has been fairly well characterized, clinically and histologically. In contrast, recurrence of blue nevi has been reported infrequently [11,12]. In a study by Harvell et al., no malignant transformation or satellitosis was detected in recurrent BN [11].

We report a unique case of a recurrent CBN accompanied by satellite lesions after excision of a CBN five years before.

## 2. Materials and Methods

**Figure 1:**
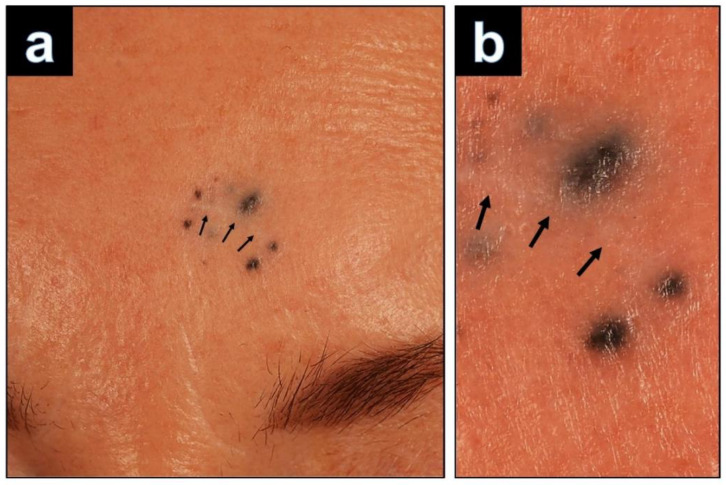
(**a**): Left forehead: at the left side of the forehead, a linear scar (black arrows) with size of 10 × 2 mm is surrounded by eight blue-black satellite lesions measuring up to 2 mm. (**b**): Magnification of the satellite lesions.

A 64-year-old woman was referred to our department with a suspected melanoma on her left forehead. Five years earlier, she had undergone excision of a blue papule at this site, which had been removed incompletely. Two years after removal the patient recognized a new pigmented lesion surrounding the scar. She noticed progression in size and pigmentation and consulted a dermatologist. She was referred to our department where she presented with a scar on the left forehead that was surrounded by eight bluish black macules (Figure 1a,b). There were no signs of lymphadenopathy by palpation and ultrasound examination. The lesion was excised with the suspected clinical diagnosis of recurrent disseminated malignant blue nevus. The tissue was formalin-fixed and paraffin-embedded, and 5-μm-thick sections were prepared for hematoxylin and eosin (H&E) staining and immunohistochemical studies. Applied antibodies were directed against Melan-A (Dako, monoclonal, Clone A103), SOX10 (Cell Marque, monoclonal, Clone EP268), HMB45 (DAKO, monoclonal, Clone HMB45), S100 (Dako, polyclonal), BAP1 (Santa Cruz Technology, polyclonal, Clone C-4) and Ki-67 (Dako, monoclonal, Clone MIB-1).

## 3. Results

**Figure 2:**
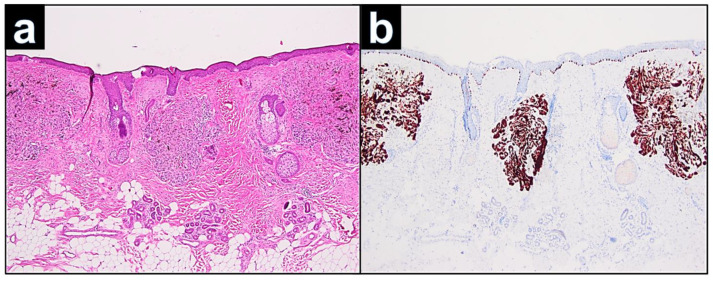
(**a**): According to the clinical findings, dermal separated nodes of the relapsed cell-rich blue nevus with ovoid and dendritic melanocytes (H&E, 40×). Inset H&E 400×. (**b**): Positivity of the melanocytes in the immunostaining with Melan-A (Melan-A, 40×) Inset Melan A × 400.

**Figure 3:**
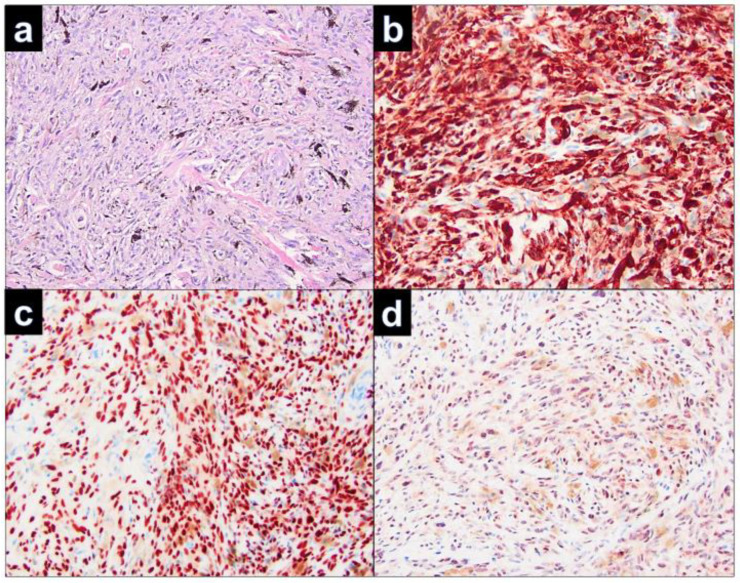
(**a**) Recurrent cellular blue cell nevus with strongly pigmented oval-shaped, spindle-shaped, and dendritic melanocytes and melanophages, occupying upper and mid dermis. (H&E, 200×). The tumor cells are diffusely positive for Melan-A (**b**) (200×), SOX10 (**c**) (200×), and BAP1 (**d**) (200×).

Histopathological examination of the relapsed cellular blue nevus showed solitary nodules in close vicinity to the scar (Figure 2a). The nodules were constituted by a nodular dermal proliferation of ovoid melanocytes, with monomorphous nuclei and inconspicuous cytoplasm, intermingled with dendritic pigmented melanocytes. At immunohistochemistry, either the ovoid or the dendritic cells were positive with Melan-A (Figure 2b). Nevus cells of the nodules were dispersed in the dermis and hyperpigmented, spindle-shaped melanocytes infiltrated among the collagen bundles. There were no features suggestive of malignancy, such as cytological atypia, atypical mitoses, or necrosis (Figure 3a). Positivity in the melanocytes with the Melan-A immunostains (Figure 3b), SOX 10 (Figure 3b), HMB45, S100, and BAP1 (Figure 3d) was seen.

**Figure 4:**
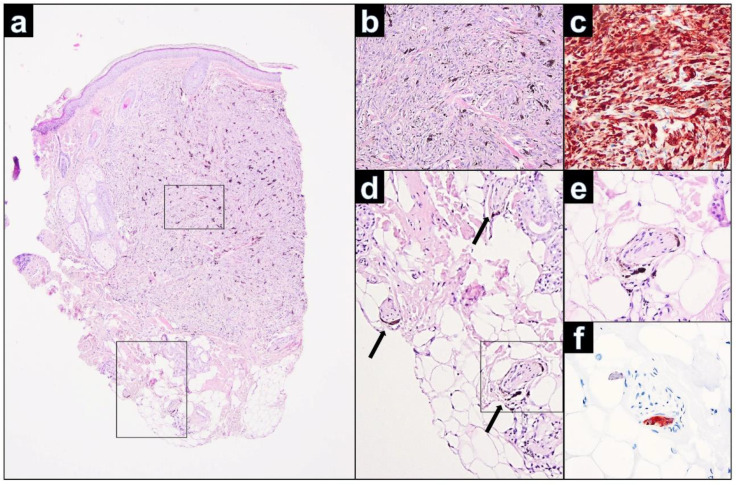
(**a**): Left forehead: primary CBN showing strongly pigmented melanocytes with dense fibrous stroma and melanophages. Cellular areas of CBN extend vertically into the deep reticular dermis (H&E, 25×). (**b**): Strongly pigmented oval-shaped, spindle-shaped, and dendritic melanocytes and melanophages, occupying the dermis. No features suggestive of malignancy were seen, such as cytological atypia, atypical mitoses, or necrosis (H&E, 200×). (**c**): The tumor cells are diffusely positive for Melan-A. (**d**): Small groups of melanocytes and melanophages colonize the endoneurium of small nerves (black arrows) (H&E, 40×). (**e**): Presence of blue nevus melanocytes in the wall of a peripheral nerve fiber (H&E, 400×). (**f**): Immunohistochemically, the melanocytes in the wall of the nerve fiber are strong positive for Melan-A (Melan-A, 400×).

On histology of the primary lesion, the CBN was symmetric and revealed spindle-shaped and dendritic, strongly pigmented melanocytes, amid dense fibrous stroma and melanophages, predominantly occupying upper and mid dermis. (Figure 4a–c). Mitoses were absent. A superficial subepidermal grenz zone without junctional involvement was present. Both the primary and the recurrent lesion showed identical infiltrates of a benign cellular blue nevus. Concentrations of the nevus cells were mainly observed in the primary lesion periappengeal and perivascular. Further, we could demonstrate melanocytes and melanophages in the wall of small peripheral nerves (Figure 4d–f).

## 4. Discussion

We performed a literature review using PubMed (search terms: blue nevus, satellitosis) to identify earlier reported cases of BN with satellitosis between 1999 and 2022. We only became aware of 10 BN cases with satellitosis, making our case the 11th report. The most important features of these cases are summarized in Table 1.

Histopathologically, only three cases were CBN. The mean age of the patients was 47.8 years, with the youngest patient being 15 and the oldest 71 years. The predilection sites for BN with satellitosis are the scalp, forehead, and the upper extremities. Tumor sizes ranged from 6.0 mm to 18.0 mm (average 10.5 mm). In almost all reports, melanoma or a malignant BN was the initially favored clinical diagnosis. In all 11 cases, it was difficult to distinguish BN with satellitosis from malignant melanoma without histological examination as satellite lesions of pigmented tumors are clinically often considered as a sign of malignant transformation.

Max Tieche first described BN in 1906 [13]. BN evolve as the result of an ectopic accumulation of melanocytes retained in the dermis during their migration from the neural crest to the epidermis. BN and related entities represent a heterogeneous group of congenital and acquired melanocytic tumors that includes dendritic (“common”) blue nevi (DBN), cellular blue nevi (CBN), and variants such as atypical cellular blue nevi (ACBN) and malignant BN/melanoma.

Desmoplastic BN is considered as another important variant of BN, which must not be confused with desmoplastic melanoma [14]. Epithelioid BN is a diagnostic challenging entity because of its rarity and histological overlap with CBN and malignant BN [15].

CBN differs from the classic DBN by its large size, cellularity, strong pigmentation, and growing pattern with subcutaneous infiltration. Additional atypical features associated with a variety of CBN histological patterns, but without clear-cut evidence of malignancy, have been referred to as ACBN. Clinically, ACBNs resemble CBNs, but histologically, they contain worrisome features, such as asymmetry, hypercellular foci, focal cytological atypia, and occasional mitoses (<2/mm^2^) [16,17]. Malignant BN is a very rare form of melanoma, arising in association with or exhibiting some morphologic similarities to BN. Pigmented epithelioid melanoytoma (previously termed animal-type melanoma) is another rare variant of cutaneous melanoma that may also mimic melanoma associated with blue nevus [18].

The variant described here, a CBN with satellite lesions mimicking malignant melanoma with cutaneous metastases, is extremely rare. The term “satellite” is usually defined as a skin cancer that has spread from the primary tumor through the lymphatic system to a distance of no more than 2 cm [19]. In benign tumors such as BN, the term “satellite” is used regardless of the dissemination route of the cells or the distance between the satellite and the primary tumor. Since satellite lesions usually occur in malignant neoplasms, the differential diagnosis of BN with satellitosis is locally advanced melanoma or malignant BN. Another differential diagnosis of BN with satellitosis is agminated blue nevus. Only a few cases of multiple, agminated, or plaque-type blue nevi have been reported [20]. In contrast to cases of BN with satellitosis, the agminated subtype is formed when bluish-pigmented lesions cluster together in a circumscript area without a central main papule or nodule [21].

The etiology and pathogenesis of satellitosis in BN is still unclear. In several studies, a spread of the melanocytes along small hair vessels and skin appendages starting from the primary lesion was observed [1,4,8]. The fact that the nevus cells were densely aggregated around the blood vessels in the main papule and in the satellite lesions implicated spreading of the nevus cells along the perivascular space to manifest clinically satellite lesions. It is therefore conceivable that, in our case, the scarred sections in the area of the pre-existing primary lesion could have served as a guide for the spread of melanocytes. Further, it is also known that nerve sheaths within BN contain dendritic melanocytes that could also migrate [22]. The primary lesion of our case showed melanocytes in the wall of small peripheral nerve fibers. We speculate that this could be another possibility of nevus cell spread in cases of BN with satellitosis, especially when CBN are involved.

Recurrent melanocytic nevus is a proliferation of melanocytes arising from a partially excised melanocytic nevus. Recognizing this phenomenon is important, because recurrent melanocytic nevi often display atypical borders and irregular pigmentation, which can lead to diagnostic confusion with melanoma. The pathologic features of recurrent nevus and melanoma are often very similar. Kelly et al. claimed that the most suspicious feature for melanoma in these cases is the growth of the lesion beyond the confines of the initial scar into the surrounding normal skin [12]. To differentiate recurrent nevus from melanoma, other diagnostic tools such as immunohistochemistry, dermoscopy, and confocal microscopy should be used in addition to standard (immuno)histologic work-up.

Recurrence of BN has rarely been reported. Harvell et al. performed a study to better define these entities [11]. Clinical recurrence is often considered to be associated with malignant transformation in BN, but a study by Harvell et al. has shown that malignant tumor progression is not necessarily the case. In the absence of necrosis, marked cytological atypia, and frequent mitotic figures, atypical morphologic parameters in BN are probably reactive and “pseudomalignant”. In our case, the recurrence of the cell-rich BN is characterized by a significant extension of melanocytes beyond the scar, but no increased cytological atypia or mitotic activity could be detected. The follow-up of our case (currently 36 months) showed an inconspicuous course without evidence of recurrence or metastasis.

In conclusions, satellitosis is a very rarely reported phenomenon in BN (and even rarer in CBN) that may lead to clinical appreciation as melanoma. On histopathologic examination of our case, we found malignant changes neither within the primary CBN nor in the recurrent CBN-associated satellite lesions. It is important to realize that a recurrent pigmented lesion with satellitosis does not necessarily represent malignant melanoma: it may rather also be an unusual manifestation of a recurrent BN.

## Figures and Tables

**Table 1:** Overview on cases of blue nevi with satellitosis reported in the literature.

Case (Reference)	Histological Diagnosis	Year of Report	Age	Sex	Localization	Size	Initial Clinical Diagnosis
1 [1]	Common blue nevus with satellitosis	1999	31	Female	Forearm	8 mm	Malignant melanoma with peripheral spread
2 [2]	Benign blue nevus with satellitosis	2000	66	Male	Scalp	18 mm	Malignant melanoma; common blue nevus
3 [3]	Benign blue nevus with satellitosis	2001	70	Male	Scalp	8 mm	Malignant melanoma; malignant blue nevus
4 [4]	Common blue nevus with satellitosis	2009	33	Female	Scalp	10 mm	Malignant melanoma;
5 [5]	Cellular blue nevus with satellitosis	2009	71	Female	Scalp	6 mm	Malignant melanoma with metastasis
6 [6]	Benign blue nevus with satellitosis	2012	70	Male	Scalp	8 mm	Not mentioned
7 [7]	Common blue nevus with satellitosis	2013	24	Male	Scalp	10 mm	Malignant melanoma; common blue nevus
8 [8]	Common blue nevus with satellitosis	2017	15	Female	Forearm	8 mm	melanoma
9 [9]	Common blue nevus with satellitosis	2018	57	Male	Forehead	16 mm	melanoma
10 [10]	Cellular blue nevus with satellitosis	2022	25	Male	Chest	22 mm	Malignant blue nevus
11	Recurrent cellular blue nevus with satellitosis	Present case	64	Female	Forehead	10 mm	Malignant melanoma with satellite metastases

## Data Availability

Not applicable.

## References

[1] Kang D., Chung K.-Y. (1999). Common blue naevus with satellite lesions: Possible perivascular dissemination resulting in a clinical resemblance to malignant melanoma. Br. J. Dermatol..

[2] del Río E., Vázquez Veiga H.A., Suárez Peñaranda J.M. (2000). Blue nevus with satellitosis mimicking malignant melanoma. Cutis.

[3] Sahin M., Demir M., Yoleri L., Can M., Ozturkcan S. (2001). Blue naevus with satellitosis mimicking malignant melanoma. J. Eur. Acad. Dermatol. Venereol..

[4] Pina S., Grenzi L., Albertini G. (2009). Cellular Blue Nevus with Satellitosis: A Possible Diagnostic Pitfall. Am. J. Dermatopathol..

[5] Knopp E., Diette K., Ko C., Lazova R. (2009). Multiple Blue Macules and Papules on the Scalp—Quiz Case. Arch. Dermatol..

[6] Lourari S., Lamant L., Viraben R., Paul C., Meyer N. (2012). Photoletter to the editor: Blue nevus with satellitosis mimicking melanoma. Contribution of dermoscopy and reflectance confocal microscopy. J. Dermatol. Case Rep..

[7] Yonei N., Kimura A., Furukawa F. (2013). Common Blue Nevus with Satellite Lesions Needs aDifferential Diagnosis from Malignant Melanoma. Case Rep. Dermatol..

[8] Oliveira A.H.K., Sotero P.D.C., Shiraishi A.F.D.M.C., Stelini R.F., Kadunc B.V., Mendes C. (2017). Blue nevus with satellitosis: Case report and literature review. An. Bras. Dermatol..

[9] Sardoy A., Bidabehere M., Gubiani M., Pinardi B. (2018). Blue Nevus with Satellite Lesions Mimicking Malignant Melanoma. Actas Dermosifiliogr..

[10] Matsuki Y., Uetake Y., Yamashita H., Yokoyama M., Suyama T., Katagiri K., Ono Y., Ito K. (2022). Case of a cellular blue nevus with multiple satellite lesions showing radial blue-white lesion: Unique dermoscopy-histopathology correlation in blue nevus with satellitosis [published online ahead of print, 2022 May 16]. J. Dermatol..

[11] Harvell J.D., White W.L. (1999). Persistent and Recurrent Blue Nevi. Am. J. Dermatopathol..

[12] Kelly J., Shen S., Pan Y., Dowling J., McLean C. (2014). Postexcisional melanocytic regrowth extending beyond the initial scar: A novel clinical sign of melanoma. Br. J. Dermatol..

[13] Tieche M. (1906). Über benigne Melanome (“Chromatophorome”) der Haut-“blaue Naevi”. Virchows Arch. F. Path. Anat..

[14] Brambullo T., Toninello P., Sonda R., Salmaso R., Sacchi D., Piaserico S., Bassetto F. (2021). A Misdiagnosed Desmoplastic Neurotropic Melanoma of the Scalp: A Challenging Case for the Pathologist and Surgeon. Acta Dermatovenerol Croat..

[15] Lee C.H., Min H.S., Park E.S., Song K.Y. (2014). A Case of Epithelioid Blue Nevus. Korean J. Pathol..

[16] Murali R., McCarthy S.W., Scolyer R.A. (2009). Blue nevi and related lesions: A review highlighting atypical and newly described variants, distinguishing features and diagnostic pitfalls. Adv. Anat. Pathol..

[17] Andrei R., Zurac S., Socoliu C., Mandisodza P., Stăniceanu F. (2015). Problems of Differential Diagnosis in Melanoma Arising from Blue Naevus. Romanian J. Intern. Med..

[18] Cazzato G., Arezzo F., Colagrande A., Cimmino A., Lettini T., Sablone S., Resta L., Ingravallo G. (2021). “Animal-Type Melanoma/Pigmented Epithelioid Melanocytoma”: History and Features of a Controversial Entity. Dermatopathology.

[19] Gershenwald J.E., Scolyer R.A., Hess K.R., Sondak V.K., Long G.V., Ross M.I., Lazar A.J., Faries M.B., Kirkwood J.M., McArthur G.A. (2017). Melanoma staging: Evidence-based changes in the American Joint Committee on Cancer eighth edition cancer staging manual. CA Cancer J. Clin..

[20] Paolino G., Didona D., Lopez T., Alesini F., Cantisani C., Richetta A.G., Soda G., Calvieri S. (2016). Agminated Blue Nevus: Two Case Reports and a Mini-review of the Literature. Acta Dermatovenerol. Croat..

[21] Lisboa A.P., Silvestre K.J., Pedreira R.L., Alves N.R.D.M., Obadia D.L., Azulay-Abulafia L. (2016). Agminated blue nevus-Case report. An. Bras. Dermatol..

[22] Misago N. (2000). The relationship between melanocytes and peripheral nerve sheath cells (Part II): Blue nevus with peripheral nerve sheath differentiation. Am. J. Dermatopathol..

